# Graphical Approach to Interpreting and Efficiently Evaluating Geminal Wavefunctions

**DOI:** 10.1002/qua.70000

**Published:** 2024-12-20

**Authors:** Michelle Richer, Taewon D. Kim, Paul W. Ayers

**Affiliations:** ^1^ Department of Chemistry Queen's University Ontario Canada; ^2^ Department of Chemistry & Chemical Biology McMaster University Ontario Canada; ^3^ Department of Chemistry and Quantum Theory Project University of Florida Gainesville Florida

**Keywords:** antisymmetric product of geminals, electron correlation, electronic structure theory, graph theory, parameterized configuration interaction, quantum chemistry, quasiparticles

## Abstract

We consider wavefunctions built from antisymmetrized products of two‐electron wavefunctions (geminals), which is arguably the simplest extension of the antisymmetrized product of one‐electron wavefunctions (orbitals) (i.e., a Slater determinant). Extensive use of geminals in wavefunctions has been limited by their high cost stemming from the many combinations of the two‐electron basis functions (orbital pairs) used to build the geminals. When evaluating the overlap of the APG wavefunction with an orthogonal Slater determinant, this cost can be interpreted as the cost of evaluating the permanent, resulting from the symmetry with respect to the interchange of orbital pairs, and the cost of assigning the occupied orbitals to the orbital pairs of the wavefunction. Focusing on the latter, we present a graphical interpretation of the Slater determinant and utilize the maximum weighted matching algorithm to estimate the combination of orbital pairs with the largest contribution to the overlap. Then, the cost due to partitioning the occupied orbitals in the overlap is reduced from 𝒪((N−1)!!) to $𝒪(N^{3}\log N)$. Computational results show that many of these combinations are not necessary to obtain an accurate solution to the wavefunction. Because the APG wavefunction is the most general of the geminal wavefunctions, this approach can be applied to any of the simpler geminal wavefunction ansätze. In fact, this approach may even be extended to generalized quasiparticle wavefunctions, opening the door to tractable wavefunctions built using components of arbitrary numbers of electrons, not just two electrons.

## Graphical abstract

Using a graphical interpretation of geminal wavefunctions, the maximum weighted matching algorithm provides an estimate for the combination of orbital pairs with the largest contributions to the wavefunction. This allows one to accurately approximate results for a fully general antisymmetric product of geminals wavefunctions with polynomial cost.

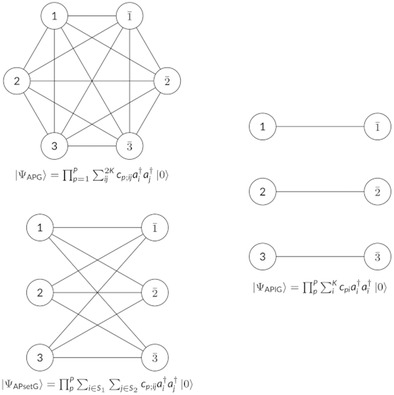

## 1 From Orbital‐Based to Geminal‐Based Wavefunctions

The simplest practical N‐electron wavefunction that satisfies the Pauli exclusion principle is a Slater determinant of spin orbitals. In most practical calculations, the spin orbitals, which are merely one‐electron wavefunctions, are expanded as a linear combination of one‐electron basis functions, which are typically chosen to approximate atomic orbitals. It is common to then optimize the molecular orbitals by minimizing the energy, resulting in the venerable Hartree‐Fock wavefunction,

(1) $$\left|\Phi_{\text{HF}}\rangle =\overset{N}{\underset{k=1}{\prod }}\;\overset{2K}{\underset{i=1}{\sum }}\;c_{ki}a_{i}^{†}|0\right\rangle$$

where N is the number of electrons, K is the number of spatial basis functions (2K is the number of spin‐basis functions), and C is constrained so that the orbitals are orthogonal and normalized [1, 2, 3, 4, 5, 6, 7, 8, 9]. We use |0⟩ to represent the vacuum state, which is typically (but not necessarily) the zero‐electron state.

The Slater determinant wavefunction represents a system of independent fermions and intrinsically omits electron correlation. The typical approach to electron correlation is to correct the Hartree‐Fock method by computing contributions from additional Slater determinants, but this approach can be very inefficient for strongly correlated systems, where the Hartree‐Fock wavefunction can be a very poor starting point (e.g., it may have a very small overlap with the exact wavefunction) and/or myriad Slater determinants may be required. This suggests an alternative approach, philosophically similar to the Hartree‐Fock method, wherein the wavefunction is constructed as an antisymmetric product of 2‐electron wavefunctions, called geminals (Obviously, the method can be extended to composite particles composed of three (ternions), four (quartets), or more electrons [10, 11].).

In analogy to the basis set expansion for orbitals, in practical calculations, geminals are usually expanded as a linear combination of two‐electron basis functions. In analogy to the Slater determinant, the simplest N‐electron geminal‐based wavefunction is an antisymmetrized product of P=N/2 geminals [12, 13, 14, 15, 16, 17, 18]. (In this paper we will assume that there are an even number of electrons in the system, but there are standard approaches for treating odd‐electron systems with geminals [19, 20, 21, 22, 23].) It is convenient to choose the two‐electron basis functions to be *orbital pairs*: antisymmetrized products of one‐electron orthonormal basis functions (i.e., orbitals).

In its most general form, the Antisymmetrized Product of Geminals (APG) uses all possible orbital pairs, indexed by p=1,2,…,P, and imposes no restrictions on their coefficients,

(2) $$\left|\Psi_{\text{APG}}\rangle =\overset{P}{\underset{p=1}{\prod }}\;\overset{2K}{\underset{ij}{\sum }}\;c_{p;ij}a_{i}^{†}a_{j}^{†}|0\right\rangle$$

In the APG wavefunction, all creation operators $a_{i}^{†}$ and $a_{j}^{†}$ can be paired with one another, regardless of their spin [10, 12, 13, 14, 15, 17, 24, 25, 26, 27, 28]. For both conceptual and computational purposes, it is convenient to rewrite the APG wavefunction as a linear combination of Slater determinants, each of which has a coefficient which is a sum of permanents [11],

(3) $$\begin{array}{ll} \left|\Psi_{\text{APG}}\right\rangle & =\underset{m\in \text{dets}}{\sum }\;\sum_{\{i_{1},j_{1},\ldots ,i_{P},j_{P}\}=m}\\ & \text{sgn}\left(\sigma (i_{1},j_{1},\ldots ,i_{P},j_{P})\right){\left|\begin{array}{ccc} c_{1;i_{1}j_{1}} & \ldots & c_{1;i_{P}j_{P}}\\ \vdots & \ddots & \vdots \\ c_{P;i_{1}j_{1}} & \ldots & c_{P;i_{P}j_{P}} \end{array}\right|}^{+}|m\rangle \end{array}$$

where m is a set of spin‐orbitals occupied in the Slater determinant, dets is the set of all Slater determinants, C is the geminal coefficient matrix whose columns correspond to the creation operator pairs,

(4) $$\sum_{\{i_{1},j_{1},\ldots ,i_{P},j_{P}\}=m}\cdots$$

denotes the sum over all possible combinations of creation operator pairs that construct the given Slater determinant, sgn(σ) is the signature of the permutation required to reorder $a_{i_{1}}^{†}a_{j_{1}}^{†}\ldots a_{i_{P}}^{†}a_{j_{P}}^{†}$ such that the indices of the creation operators are increasing, and

(5) $${\left|\begin{array}{lll} c_{1;i_{1}j_{1}} & \ldots & c_{1;i_{P}j_{P}}\\ \vdots & \ddots & \vdots \\ c_{P;i_{1}j_{1}} & \ldots & c_{P;i_{P}j_{P}} \end{array}\right|}^{+}=\underset{\tau \in S_{P}}{\sum }\;c_{1;i_{\tau (1)}j_{\tau (1)}}\ldots c_{P;i_{\tau (P)}j_{\tau (P)}}$$

is a permanent. In Equation (5), $\tau \in S_{P}$ denotes the sum over all permutations τ. We will refer to the creation operators as occupied spin orbitals, creation operator pairs as orbital pairs, and the combinations of creation operator pairs as pairing schemes.

The preceding equation makes it easy to place APG within the more general framework of Flexible Ansatze for N‐electron Configuration Interaction (FANCI) wavefunctions [11, 29, 30]. Specifically, the overlap of the APG wavefunction with a Slater determinant, m, is

(6) $$\begin{array}{ll} \left\langle m|\Psi_{\text{APG}}\right\rangle & =\sum_{\{i_{1},j_{1},\ldots ,i_{P},j_{P}\}=m}\\ & \text{sgn}\left(\sigma (i_{1},j_{1},\ldots ,i_{P},j_{P})\right){\left|\begin{array}{ccc} c_{1;i_{1}j_{1}} & \ldots & c_{1;i_{P}j_{P}}\\ \vdots & \ddots & \vdots \\ c_{P;i_{1}j_{1}} & \ldots & c_{P;i_{P}j_{P}} \end{array}\right|}^{+} \end{array}$$

Each term in the overlap expression corresponds to a combination of the orbital pairs (pairing scheme) to form the given Slater determinant and each term in the permanent expression corresponds to an ordering of the orbital pairs from the given pairing scheme. These two components of the APG make it prohibitively expensive: (1) the number of terms in the overlap (Equation 6) increases double factorially with the number of electrons, (2P−1)!!, and (2) the cost of evaluating the permanent increases factorially with the number of electron pairs, P!.

Different geminal wavefunctions try to offset this computation cost by truncating the two‐electron (orbital pair) basis set and by constraining the geminal coefficients [19, 22, 29, 31, 32, 33, 34, 35, 36, 37, 38, 39, 40, 41, 42, 43, 44, 45, 46, 47, 48, 49, 50, 51, 52, 53, 54, 55, 56, 57, 58]. By removing orbital pairs from the geminals, the number of terms in the sum (4) decreases. For example, in the APsetG wavefunction, only the creation operators of one set, $S_{1}$ can be paired with the creation operators of a second disjoint set, $S_{2}$ [19].

(7) $$\left|\Psi_{\text{APsetG}}\rangle =\overset{P}{\underset{p}{\prod }}\;\underset{i\in S_{1}}{\sum }\;\underset{j\in S_{2}}{\sum }\;c_{p;ij}a_{i}^{†}a_{j}^{†}|0\right\rangle$$

In the spin‐restricted Antisymmetrized Product of Interacting Geminals (APIG) wavefunction, only the creation operators that correspond to the same spatial orbital can be paired with one another [56].

(8) $$\left|\Psi_{\text{APIG}}\rangle =\overset{P}{\underset{p}{\prod }}\;\overset{K}{\underset{i}{\sum }}\;c_{pi}a_{i}^{†}a_{\bar{ı}}^{†}|0\right\rangle$$

Here i is the index for the spatial orbital with spin alpha and $\bar{ı}$ is the index for the same spatial orbital with spin beta. Both APsetG and APIG wavefunctions limit the number of terms in the overlap (Equation 6) by truncating the orbital pairs: fewer orbital pairs results in fewer pairing schemes. Specifically, APsetG reduces the terms in the sum (4) to P!; in APIG, there is only a single term in the sum.

In addition to truncating the orbital pairs, we can apply constraints to the geminal coefficients [29, 59]. For example, the Antisymmetrized Product of 1‐reference orbital Geminals (AP1roG) wavefunction is an approximation to the APIG wavefunction where the first block of the coefficient matrix is assigned as the identity matrix [59]:

(9) $$\left|\Psi_{\mathrm{AP1roG}}\rangle =\overset{P}{\underset{p}{\prod }}\left(a_{p}^{†}a_{\bar{p}}^{†}+\overset{K}{\underset{i=P+1}{\sum }}\;c_{pi}a_{i}^{†}a_{\bar{ı}}^{†}\right)|0\right\rangle$$

In this wavefunction, each geminal is dominated by a spatial orbital occupied in the reference Slater determinant. The cost of its overlap with a selected Slater determinant is 𝒪(m!) where m is the order of excitation of the selected Slater determinant with respect to the reference determinant. Unlike the AP1roG wavefunction, which approximates a portion of the APIG coefficient matrix, the Antisymmetrized Product of rank‐2 Geminals (APr2G) approximates the entire matrix as a Cauchy matrix, whose permanent can be reduced to a quotient of two determinants [21, 60, 61]. Then, the cost of the overlap reduces to that of computing the determinants, which is $𝒪(P^{3})$. Since these approximations apply only to the geminal coefficients, they can be applied to any geminal wavefunctions. In this article, however, we focus on developing new methodologies for selecting orbital pairs for the APG wavefunction and keeping the coefficients unconstrained.

## 2 From Geminal‐Based Wavefunctions to Graphs

Unlike the APIG wavefunction, the APsetG and APG wavefunctions can represent the same Slater determinant with different pairing schemes. For example, suppose we have the Slater determinant with occupied spin orbitals $\left(1,2,\bar{1},\bar{2}\right)$, where spin orbitals i and $\bar{ı}$ correspond to the alpha and beta spin parts of the ith spatial orbital. In the APG wavefunction, there are three different pairing schemes: $\left(1,\bar{1}\right)$ and $\left(2,\bar{2}\right)$; $\left(1,\bar{2}\right)$ and $\left(2,\bar{1}\right)$; and (1,2) and $\left(\bar{1},\bar{2}\right)$. The APsetG wavefunction has two pairing schemes: $\left(1,\bar{1}\right)$ and $\left(2,\bar{2}\right)$; and $\left(1,\bar{2}\right)$ and $\left(2,\bar{1}\right)$. The APIG wavefunction has just one pairing scheme: $\left(1,\bar{1}\right)$ and $\left(2,\bar{2}\right)$. Each pairing scheme corresponds to a term in the overlap; each term requires the evaluation of a permanent.

Identifying the pairing schemes associated with a given Slater determinant is analogous to dividing its occupied spin orbitals into disjoint subsets containing two elements. In other words, a pairing scheme is a set of subsets of two spin orbitals. Consistent with the Pauli exclusion principle, the subsets are all disjoint; the union of the subsets equals the set of occupied spin orbitals in the given Slater determinant.

**FIGURE 1:**
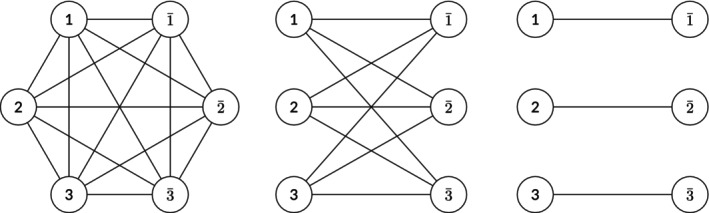
Graphs to describe the overlap of Slater determinants with occupied spin orbitals $\left(1,\bar{1},2,\bar{2},3,\bar{3}\right)$: APG (left), APsetG (center), and APIG (right) wavefunctions.

The process of finding the pairing schemes can be simplified by interpreting the occupied spin‐orbitals and subsets/orbital pairs as vertices and edges of a graph, respectively. For example, the graph for the APG wavefunction is a complete graph since all orbital pairs can be used. The graph for the APsetG wavefunction, on the other hand, is a complete bipartite graph because only the orbitals from one set can be paired with the orbitals from a disjoint set. The graph for the APIG wavefunction is a perfect matching. The graphs that correspond to the overlaps of the APG, APsetG, and APIG wavefunctions with the Slater determinant with occupied spin‐orbitals labeled by $\left\{1,\bar{1},2,\bar{2},3,\bar{3}\right\}$ are shown in Figure 1; the corresponding mathematical expressions are shown in Appendix A.

If the occupied spin orbitals and the allowed orbital pairs are represented as a graph, then each pairing scheme is a perfect matching of this graph. A perfect matching is a set of edges of a graph with no shared end points that contain all of the vertices of a graph; a pairing scheme is a set of orbital pairs with no shared orbitals that contains all of the occupied spin orbitals of a Slater determinant. Then, the sum over the pairing schemes within the overlap is equivalent to the sum over the perfect matchings of the corresponding graph. In the case of the APG wavefunction, there are $\left(2P-1)!!=(2P)!/(P!2^{P}\right)$ perfect matchings of the corresponding complete graph of 2P vertices. The number of pairing schemes grows explosively as the number of vertices/electrons increases, and the overlap becomes intractable to compute. In contrast, at least for systems of chemical relevance, it seems the APIG wavefunction is a nearly exact approximation of the Doubly Occupied Configuration Interaction (DOCI) wavefunction [62, 63, 64, 65, 66, 67, 68, 69, 70, 71, 72, 73], despite using only one pairing scheme per Slater determinant [17, 59, 74]. This suggests that not all pairing schemes are needed and that some pairing schemes are more important than others; many terms in the overlap may be negligibly small and thus can be ignored.

The goal of this work is to develop an algorithm that systematically selects the most important pairing schemes (discarding the rest) for any given Slater determinant. Since the number of pairing schemes increases exponentially with the number of electrons, this algorithm cannot consider each pairing scheme. Of course, the orbital pairs can be truncated from the geminal wavefunction, as in the APIG wavefunction, where orbital pairs are truncated according to chemical intuition. However, the methods based on heuristics often break down when the assumptions used are no longer valid for the system under study. For instance, the spin‐restricted APIG wavefunction assumes that alpha‐beta spin orbitals associated with a common spatial orbital are the most important pair, and that other orbital pairs are not significant. When the system requires significant contributions from non‐seniority zero Slater determinants, the truncated orbital pairs become more important and the APIG wavefunction is unable to accurately describe the system.

Fortunately, there already exist algorithms that find the perfect matching with the largest weights from a graph in polynomial time. In this article, we use the maximum weighted matching algorithm based on the Edmonds' Blossom algorithm [75, 76, 77]. Though the specifics of this maximum weighted matching algorithm are deferred to the references [75, 76, 77], this algorithm uses the adjacency matrix, a matrix that embodies the connectivity of the graph and the weights of the edges, to systematically contract the graph and iteratively find the (augmenting) path whose sum of weights is maximized. The cost of the Blossom algorithm is 𝒪(N2NlogN)=𝒪(N3logN) for a complete graph with N vertices.

By assigning the weights of the edges so that they correspond to the contributions of the orbital pairs to a pairing scheme, then using the Blossom algorithm, we can find the pairing scheme with the largest contribution to the overlap. In the next section, we discuss how to assign the edge weights so that they correspond to the importance of the orbital pairs. Then, in Section 4, we present various strategies for predicting the next most significant pairing schemes using only the weighted maximum matching algorithm. After presenting our test systems in Section 5, we present our numerical results in Section 6.

## 3 Orbital Pair Contribution

Recall that the Blossom algorithm finds the set of edges (orbital pairs) that maximizes the sum of the edge weights. This means that we need to define the weights of the orbital pairs, $\left\{w_{i_{1}j_{1}}\ldots w_{i_{P}j_{P}}\right\}$, such that their sum has the same *ordering* as the size of the corresponding permanents,

(10) $${\left|\begin{array}{lll} c_{1;i_{1}j_{1}} & \ldots & c_{1;i_{P}j_{P}}\\ \vdots & \ddots & \vdots \\ c_{P;i_{1}j_{1}} & \ldots & c_{P;i_{P}j_{P}} \end{array}\right|}^{+}$$

Choosing weights for the orbital‐pairing edges with this property ensures that the maximum weighted matching will produce the pairing schemes with the largest permanents. Because the permanent is expensive to compute, we need to approximate it with an upper bound. One possibility is the Hadamard‐like inequalities for the absolute value of permanents [78]:

(11) $$\text{abs}(|C{|}^{+})\leq \frac{P!}{P^{P/2}}\overset{P}{\underset{j=1}{\prod }}|c_{j}{|}_{2}$$

and

(12) $$\text{abs}(|C{|}^{+})\leq \overset{P}{\underset{j=1}{\prod }}|c_{j}{|}_{1}$$

where N is the number of columns and $c_{j}$ is the jth column of C, and

(13) $$|c_{j}{|}_{q}={\left(\sum_{i=1}^{P}\;|c_{ij}{|}^{q}\right)}^{\frac{1}{q}}$$

denotes is the $\ell_{q}$ norm. While both inequalities are tight, the scalar factor $\frac{N!}{N^{N/2}}$ increases very quickly with the size of the matrix, so for our application we choose the second inequality, even though $|c_{j}{|}_{2}\leq |c_{j}{|}_{1}$. We provide an elementary proof of Equation (12) in Appendix B.

Using Inequality (12), the importance of the pairing schemes can be efficiently compared without evaluating the permanents. According to Equation (3), the orbital pair is represented by a column of the geminal coefficient matrix and the weight of the orbital pair is the $\ell_{1}$‐norm of the corresponding column. However, the weight of the matching used in the matching algorithm is the sum of the weights of the edges, rather than their product. To directly utilize inequality (12), we take its logarithm. We observed numerical issues when the $\ell_{1}$‐norm of a column was very small, so weights below a threshold, τ, are set to zero, and, for consistency, the remaining weights are shifted by the same threshold:

(14) $$w_{ij}=\left\{\begin{array}{ll} 0\; & \text{if}\;\overset{P}{\underset{p=1}{\sum }}|C_{p;ij}|\leq \tau \\ \log\left(\overset{P}{\underset{p=1}{\sum }}|C_{p;ij}|\right)-\log(\tau )\; & \text{otherwise} \end{array}\right.$$

Here $w_{ij}$ denotes the weight of the edge that corresponds to pairing spin‐orbital i with spin‐orbital j. Using these weights, the maximum weighted matching algorithm will produce a pairing scheme that has the largest upper bound to the corresponding permanent. We propose that the ordering of the pairing schemes by the presented upper bound (Equation 12) is similar to the ordering by the permanent values but this is less important, because we can evaluate all the contributions from permanents larger than a certain threshold using the upper bound (12). One could also use the $\ell_{2}$ norm in Equation (14)—or, indeed, referring to reference [78], any $\ell_{p}$ norm—because the prefactor in Equation (11) does not affect the predicted *order* of importance of the permanents. We chose the $\ell_{1}$ norm because of its simplicity and because our intuition suggested it will be the tightest bound in this situation. Specifically, we typically use intermediate normalization, and, presuming that the reference Slater determinant has the largest coefficient, none of the orbital pairings give a weighting that exceeds 1. (Even if this were not true, one could always adjust the normalization so that it were.) For large systems P≫1, the (very large) prefactor in Equation (11) seemingly leads to a very weak upper bound.

## 4 Algorithm

When the sum over orbital pairing schemes in APG or APsetG (cf. Equation (3)) is truncated to one pairing scheme, we can find the pairing scheme with the largest upper bound of the permanent using the Blossom algorithm. To find the pairing schemes with the next largest weights, the algorithm for finding K‐best perfect matchings can be used [79]. This algorithm involves systematically removing one or more edges of the matching from the graph and applies the weighted matching algorithm to the pruned graph. An arbitrary number of pairing schemes can be obtained in the order of decreasing weight using the K‐best perfect matching algorithm. The cost of this algorithm scales linearly with the number of selected pairing schemes.

When using the K‐best perfect matching algorithm to evaluate Slater determinant overlaps with a geminal wavefunction, only the orbital pairs whose coefficients have an $\ell_{1}$‐norm greater than the thresholds are used. If the $\ell_{1}$‐norm drops below the given threshold, then this orbital pair will not longer contribute to the wavefunction for the remainder of the optimization. In addition, it is possible that an orbital pair does not contribute to the wavefunction despite having an $\ell_{1}$‐norm above the threshold because all the pairing schemes to which it belongs have been truncated. To avoid such situations, noise can be added to the coefficients during the optimization process. The coefficients to which the noise is added can be determined according to some heuristic or probability distribution. However, these more sophisticated algorithms have not been explored in this initial study.

## 5 Computational Protocol

**FIGURE 2:**
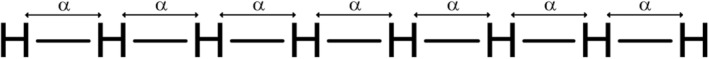
Linear H_8_ chain: α∈ {0.6, 0.7, 0.8, 0.9, 1, 1.1, 1.2, 1.3, 1.4, 1.5, 1.6, 1.7, 1.8, 1.9, 2, 2.25, 2.5, 3, 4} Angstroms.

**FIGURE 3:**
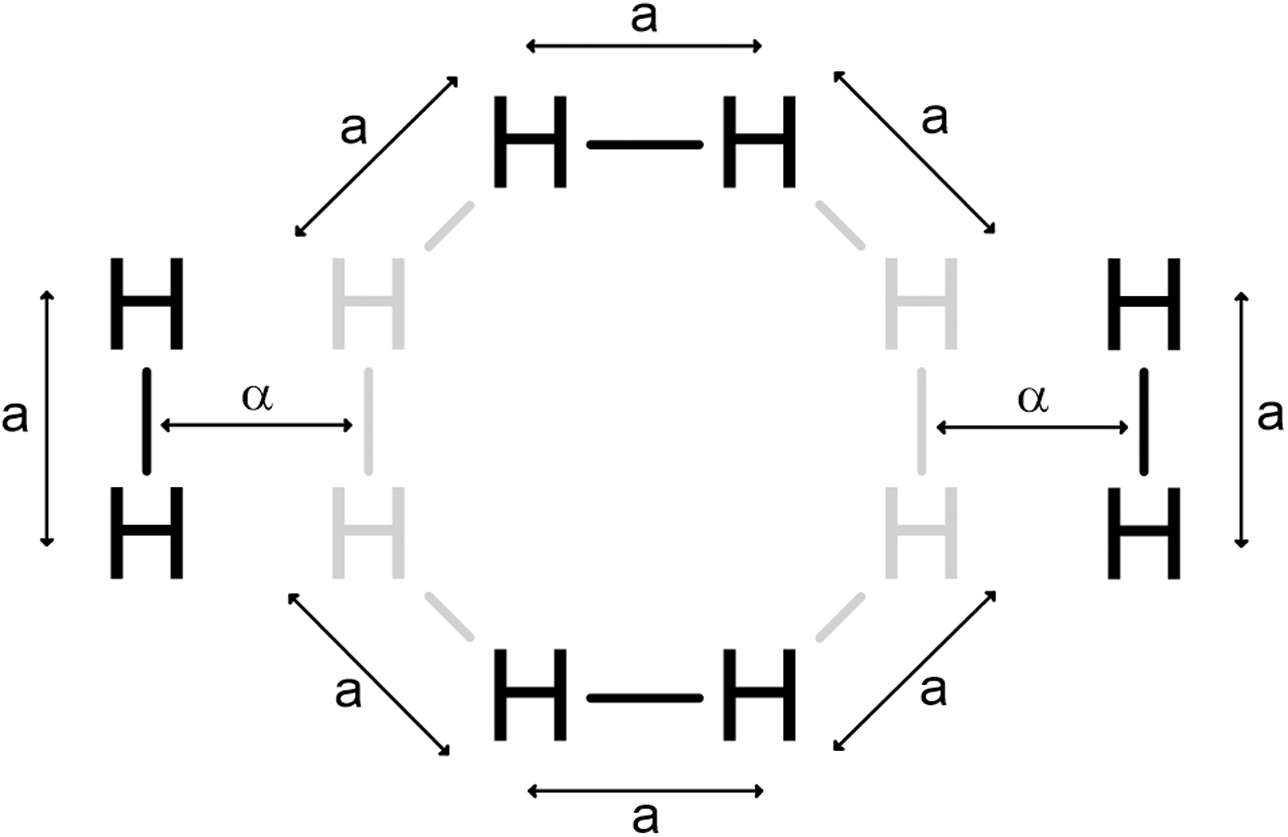
Octagonal H_8_: a = 2 a.u., α∈ {0, 0.0001, 0.001, 0.003, 0.006, 0.01, 0.03, 0.06, 0.1, 0.5, 1} a.u.

To test the accuracy of the aforementioned method for selecting the most important perfect matchings, we studied the symmetric dissociation of a H_8_ chain (Figure 2) [59, 80] and the symmetric stretch of a ring of four Hydrogen molecules (Figure 3) [81]; in both cases we used a minimal (ANO‐1s) basis set [82]. We chose these systems because accurate full configuration interaction (FCI) reference data is available and because, for these small systems, the exact evaluation of the overlap (Equation 6) is feasible. Though these systems have few electrons and basis functions, they are challenging to model with traditional electronic structure methods because they are very strongly multiconfigurational, as many bonds are being broken simultaneously and there are many nearly degenerate orbitals near the Fermi level.

The Restricted HF wavefunction was obtained from Gaussian 2016 [83] and the 1‐ and 2‐electron integrals were obtained from the gbasis [84] module of HORTON [85, 86]. The FCI wavefunction was computed using PySCF [87]. All other wavefunctions were implemented and obtained using our own FanCI software, Fanpy [88] and PyCI [89]. The Blossom algorithm was obtained from the networkx package [90]. However, rather than using the efficient K‐best perfect matching algorithm, in this initial study we obtained the K‐best pairing schemes in a brute force fashion by explicitly ordering the weights of the pairing schemes. The threshold for computing the weights of the orbital pairs in Equation (14) was $\tau =10^{-4}$.

## 6 Results and Discussion

We considered the following wavefunctions: Ap1roG, APIG, DOCI, FCI, and APG; each wavefunction was computed both with and without truncation. With the exception of the FCI wavefunction, the orbitals of these wavefunctions were optimized. The truncated APG wavefunction will be denoted as ‐Kps, where K denotes that only the terms in the sum (4) corresponding to the K‐best perfect matchings are included. We built the APG‐Kps wavefunctions using the APIG‐optimized orbitals.

**FIGURE 4:**
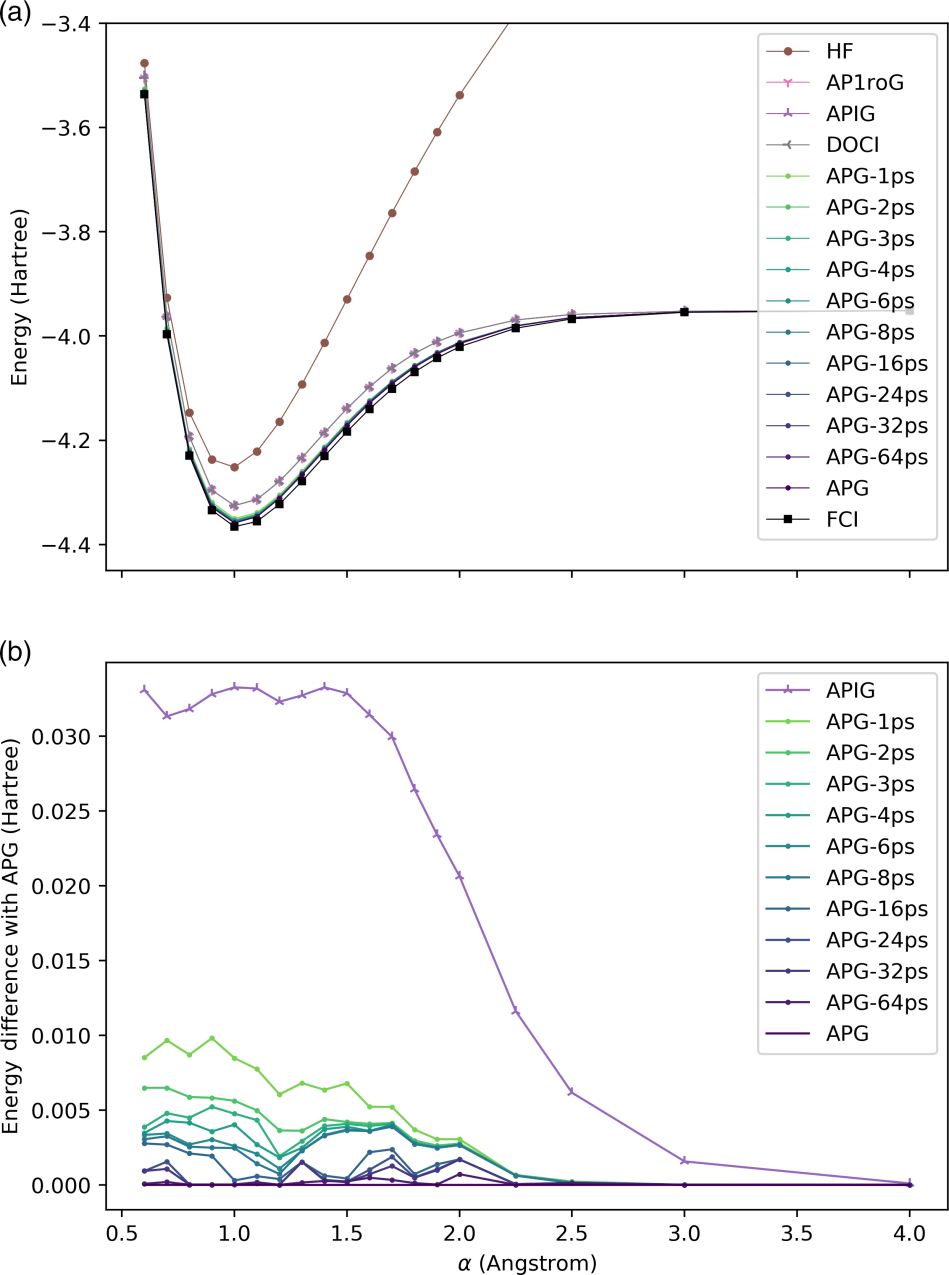
(a) Energies and (b) energy differences relative to APG in the H_8_ chain; see Figure 2.

**FIGURE 5:**
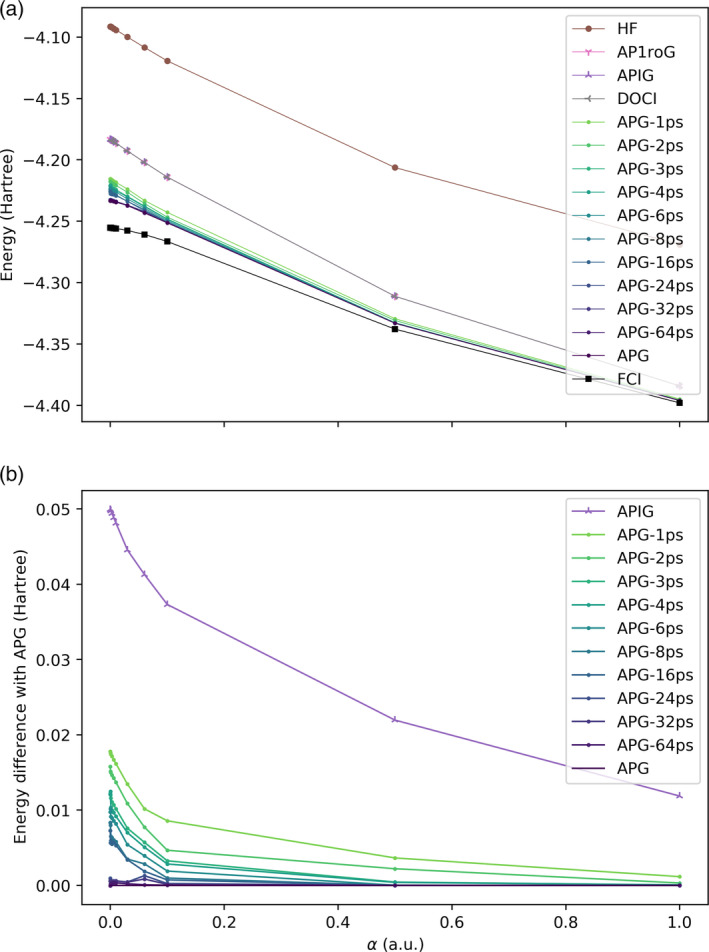
(a) Energies and (b) energy differences relative to APG in the H_8_ ring; see Figure 3.

The energies of these wavefunctions and the relative energies of the truncated APG‐Kps geminal wavefunctions with respect to the APG wavefunction are shown in Figures 4 and 5. The Hartree‐Fock energy is very inaccurate in all cases, confirming the multiconfigurational nature of these test systems. The seniority‐zero wavefunctions (AP1roG, APIG, and DOCI) are all qualitatively correct and visually indistinguishable from one another, confirming once again that the AP1roG wavefunction is a very good approximation to the APIG wavefunction for systems with repulsive interparticle interactions and that the APIG wavefunction is, in turn, an excellent approximation to the DOCI wavefunction [59]. However, the seniority‐zero methods are systematically above the FCI curves due to the absence of dynamic correlation, which is nonnegligible even in this small basis set, especially for small internuclear distances. Dynamic correlation within electron pairs (but not the more challenging intergeminal correlation between electron pairs) is included in the APG wavefunction, which is systematically more accurate than AP1roG and APIG. Note that unlike seniority‐zero methods, which are qualitatively incorrect near α=0 for the H_8_ ring because they predict a conical intersection, APG apparently models the avoided crossing at α=0 qualitatively correctly.

APG is very expensive, so it is reassuring that the energies from the APG‐Kps methods quickly approach the energy from APG as K increases. APG‐Kps converges to the APG much faster for the H_8_ chain than the H_8_ ring, probably because in a ring the atoms are closer together, and there are more important pairings. It is conceivable that the convergence would be faster if we optimized the orbitals in the APG‐Kps wavefunction [55, 74]. Nonetheless, even when K=64, a substantial portion of the 7!!=105 pairing schemes in the full APG wavefunction have been omitted.

It is remarkable that even though APG‐1ps uses a single pairing scheme like APIG, its energy error (relative to exact APG) is about three times smaller. This is because, unlike APIG, where the pairing scheme is preassigned and universal and can only be changed by optimizing the spatial orbitals, the pairing scheme in APG‐1ps is dynamically selected based on APG coefficients and the occupied orbitals (different pairing schemes will be used for the projection on different Slater determinants). Indeed, the energy error of APG‐1ps relative to APG is comparable to the energy error of APG relative to FCI. The computational cost of APG‐Kps grows linearly with K, so the cost of including additional pairing schemes is not prohibitive.

The disadvantage of APG‐Kps is that there are derivative discontinuities in the energy when the elements of the set of K‐best pairing schemes change. This is most visible in Figure 4b, where the errors in APG‐Kps are a little erratic, as compared to the smoother errors in APIG. For both APIG and APG‐Kps, some of the wiggles may be attributed to the presence of many near‐degenerate local minima in the orbital optimization. The magnitude, if not the number, of derivative discontinuities in APG‐Kps can be decreased as much as desired by increasing the number of pairing schemes. One could also attempt to mitigate the derivative discontinuities and improve the accuracy of APG‐Kps by extrapolating the result of a series of APG‐Kps calculations to APG (K=P!!). Extrapolation with respect to K is probably problematic (notice how sensitive the rate/pattern of convergence with respect to K depends on geometry, but extrapolation with respect to the magnitude of the smallest included determinant or the threshold in Equation (14) would be sensible.

APG‐Kps is also useful for optimizing the geminal coefficients in APG. For example, one can start by loosely converging the APG‐Kps coefficients for a small number of pairing schemes and then systematically increase K as the convergence threshold is tightened, using the previous solution as an accurate initial guess. Similarly, the threshold for the weights of the edges (Equation 14) can be systematically tightened during the convergence procedure. We have not, however, explored these more complicated algorithms here, as their primary utility would be for larger systems where brute‐force APG calculations are intractable.

Even greater gains can be obtained by making an analogy between selecting pairing schemes in APG‐Kps and choosing determinants in selected configuration interaction (CI) methods. In this analogy, the APG wavefunction is analogous to FCI. One could remove pairing schemes from the APG based on certain system‐independent rules; this gives approaches like APIG and APsetG, which are analogous to systematically truncated CI wavefunctions like CISD, CASSCF, DOCI, etc. [91, 92, 93, 94, 95]. Though these wavefunctions are much more affordable, they lack critical correlations. APG‐Kps is analogous to selected‐CI algorithms like CIPSI [96] and HCI [97], where one selects configurations based on the estimated size of their coefficient [96, 97, 98, 99, 100, 101, 102, 103, 104, 105, 106, 107, 108]. To further extend APG‐Kps, one may be inspired by the FCI‐QMC approach [109, 110, 111, 112, 113, 114, 115, 116, 117, 118, 119, 120, 121, 122], and selecting the pairing schemes by sampling from a probability distribution instead of by using a deterministic algorithm as we have done here.

Another possible improvement is to use faster methods for selecting the K‐best pairing schemes. State‐of‐the‐art weighted matching algorithms have a cost of $𝒪(N^{2}\sqrt{N})$ for the exact algorithm [123, 124] and $𝒪(N^{2}\epsilon^{-1}\log\epsilon^{-1})$ for the (1−ϵ) approximate algorithm [125]. The approximate algorithm returns a pairing scheme with a weight that is within a factor of (1−ϵ) of optimal. These matching algorithms are not limited to complete graphs, so they can be used for any geminal wavefunction, including APsetG.

These algorithmic improvements, however, are limited by the APG wavefunction. The APG has some desirable features (size consistency) but it is not exact for systems with more than two electrons because it neglects the correlations between geminals. We envision that the primary importance of this work is to make it more tractable to generate benchmark APG numbers, thereby providing a standard against which more approximate geminal methods may be assessed.

To go beyond APG, one must include intergeminal correlations. One can achieve this by adding basis functions with different numbers of electrons to form a generalized quasiparticle wavefunction [10, 11]. Just as the pairing schemes correspond to the perfect matches, each term in the overlap of the generalized quasiparticle wave function corresponds to a partition of the electrons into subsets. In fact, a geminal wavefunction is a special case of the generalized quasiparticle wavefunctions and a perfect matching are special cases of partitions. Adding, for example, 3‐ and 4‐electron basis functions results in an exponential increase in the number of terms in the overlap because there are many more ways to partition the given occupied orbitals into the available components. This wavefunction will be significantly more expensive to compute than the APG wavefunction, which was already intractable. However, dynamically selecting the pairing schemes in the APG wavefunction produced results comparable to those using all pairing schemes, despite being much cheaper. Similarly, it may be possible to omit many partitions within the overlap of the generalized quasiparticle wavefunction without a significant impact on the wavefunction. This approach allows the basis functions to be built up systematically, starting from the optimization of the wavefunction with the smaller body terms, such as 1‐ and 2‐electron basis functions, and subsequently adding the larger body terms. This would represent a pathway whereby one selects orbital groupings and systematically converges to the exact wavefunction, in analogy with various selected/stochastic CI algorithms.

Although this article focused on reducing the number of pairing schemes, reducing the cost of permanent evaluation is arguably even more critical for producing tractable geminal (and quasiparticle) wavefunctions. The 1‐reference‐orbital and rank‐2 approximations used in AP1roG and APr2G are clearly generalizable to the APG wavefunction. Preliminary indications are that this strategy can give excellent results [29]. These important practical embellishments on the general strategy we present here are an important target for future work. To give an idea of the types of performance that can be achieved, if the 1‐reference‐orbital structure for evaluating the permanent (analogous to AP1roG), then APG‐Kps‐1ro would have a computational cost that was about K times that of AP1roG, times the cost of determining the perfect matchings that need to be computed. The computational scaling of APG‐Kps‐1ro is thus the same as CCSD, but unlike CCSD, APG‐Kps‐1ro is applicable to strongly correlated systems.

## 7 Summary

As the number of electrons, N, in a system increases, the antisymmetrized product of geminals (APG) becomes an increasingly intractable wavefunction form. For example, to evaluate APG using the projected Schrödinger equation, one needs to sum over all (N−1)!! possible pairing schemes (the outer sum in Equation (6)) and evaluate a permanent for each pairing scheme. Our intuition and our computational results suggest that only a tiny fraction of the pairing schemes are quantitatively important. To exploit this, we derived an efficient upper bound on the contribution of any given permanent and then used the link between optimal pairing schemes and algorithms to select the K‐best perfect matching of weighted graphs to select the K most important pairing schemes. (Alternatively, we could neglect the pairing schemes whose contributions were guaranteed to be less than a given threshold.) The APG‐1ps method, where only a single pairing scheme is chosen, has essentially the same computational cost as the venerable APIG method but recovers about 2/3 of the gap in correlation energy between the APIG and APG. As additional pairing schemes are included, the APG‐Kps methods quickly converge to the exact APG‐(N−1)!!ps results. The APG‐Kps method opens the possibility for sophisticated optimization algorithms that select the most appropriate pairing schemes and the corresponding wavefunction parameters, as well as extensions to more general quasiparticle wavefunctions. When coupled with efficient parameterizations of the geminal coefficients that reduce the cost of evaluating the permanent, the APG‐Kps strategy opens up new possibilities for wavefunctions that are not only reliable for strongly correlated molecules but also computationally tractable, chemically interpretable, and numerically robust.

## Author Contributions

All authors contributed to the formulation of the project, mathematical derivations, and composition of the manuscript. In addition, Taewon D. Kim and Michelle Richer wrote software and performed calculations.

## Disclosure

The authors have nothing to report.

## Conflicts of Interest

The authors declare no potential conflicts of interest.

## Data Availability

The data that support the findings of this study are available from the corresponding author upon reasonable request.

## Appendix A Explicit Equations Corresponding to Figure 1

Suppose a Slater determinant has occupied spin orbitals $\left(1,2,3,\bar{1},\bar{2},\bar{3}\right)$. The explicit equation for the overlap of the APG wavefunction with this Slater determinant is:

(A1)
$$\begin{array}{ll} & \langle 123\bar{1}\;\bar{2}\;\bar{3}|\Psi_{\text{APG}}\rangle =\sum_{\left\{i_{1},j_{1},\ldots ,i_{3},j_{3}\}=\{1,2,3,\bar{1},\bar{2},\bar{3}\right\}}\text{sgn}\left(\sigma (i_{1},j_{1},\ldots ,i_{3},j_{3})\right)\\ & {\left|\begin{array}{ccc} c_{1;i_{1}j_{1}} & c_{1;i_{2}j_{2}} & c_{1;i_{3}j_{3}}\\ c_{2;i_{1}j_{1}} & c_{2;i_{2}j_{2}} & c_{2;i_{3}j_{3}}\\ c_{3;i_{1}j_{1}} & c_{3;i_{2}j_{2}} & c_{3;i_{3}j_{3}} \end{array}\right|}^{+}\\ & \;={\left|\begin{array}{ccc} c_{1;12} & c_{1;3\bar{1}} & c_{1;\bar{2}\;\bar{3}}\\ c_{2;12} & c_{2;3\bar{1}} & c_{2;\bar{2}\;\bar{3}}\\ c_{3;12} & c_{3;3\bar{1}} & c_{3;\bar{2}\;\bar{3}} \end{array}\right|}^{+}-{\left|\begin{array}{ccc} c_{1;12} & c_{1;3\bar{2}} & c_{1;\bar{1}\;\bar{3}}\\ c_{2;12} & c_{2;3\bar{2}} & c_{2;\bar{1}\;\bar{3}}\\ c_{3;12} & c_{3;3\bar{2}} & c_{3;\bar{1}\;\bar{3}} \end{array}\right|}^{+}\\ & \;+{\left|\begin{array}{ccc} c_{1;12} & c_{1;3\bar{3}} & c_{1;\bar{1}\;\bar{2}}\\ c_{2;12} & c_{2;3\bar{3}} & c_{2;\bar{1}\;\bar{2}}\\ c_{3;12} & c_{3;3\bar{3}} & c_{3;\bar{1}\;\bar{2}} \end{array}\right|}^{+}-{\left|\begin{array}{ccc} c_{1;13} & c_{1;2\bar{1}} & c_{1;\bar{2}\;\bar{3}}\\ c_{2;13} & c_{2;2\bar{1}} & c_{2;\bar{2}\;\bar{3}}\\ c_{3;13} & c_{3;2\bar{1}} & c_{3;\bar{2}\;\bar{3}} \end{array}\right|}^{+}\\ & \;+{\left|\begin{array}{ccc} c_{1;13} & c_{1;2\bar{2}} & c_{1;\bar{1}\;\bar{3}}\\ c_{2;13} & c_{2;2\bar{2}} & c_{2;\bar{1}\;\bar{3}}\\ c_{3;13} & c_{3;2\bar{2}} & c_{3;\bar{1}\;\bar{3}} \end{array}\right|}^{+}-{\left|\begin{array}{ccc} c_{1;13} & c_{1;2\bar{3}} & c_{1;\bar{1}\;\bar{2}}\\ c_{2;13} & c_{2;2\bar{3}} & c_{2;\bar{1}\;\bar{2}}\\ c_{3;13} & c_{3;2\bar{3}} & c_{3;\bar{1}\;\bar{2}} \end{array}\right|}^{+}\\ & \;+{\left|\begin{array}{ccc} c_{1;1\bar{1}} & c_{1;23} & c_{1;\bar{2}\;\bar{3}}\\ c_{2;1\bar{1}} & c_{2;23} & c_{2;\bar{2}\;\bar{3}}\\ c_{3;1\bar{1}} & c_{3;23} & c_{3;\bar{2}\;\bar{3}} \end{array}\right|}^{+}-{\left|\begin{array}{ccc} c_{1;1\bar{1}} & c_{1;2\bar{2}} & c_{1;3\bar{3}}\\ c_{2;1\bar{1}} & c_{2;2\bar{2}} & c_{2;3\bar{3}}\\ c_{3;1\bar{1}} & c_{3;2\bar{2}} & c_{3;3\bar{3}} \end{array}\right|}^{+}\\ & \;+{\left|\begin{array}{ccc} c_{1;1\bar{1}} & c_{1;2\bar{3}} & c_{1;3\bar{2}}\\ c_{2;1\bar{1}} & c_{2;2\bar{3}} & c_{2;3\bar{2}}\\ c_{3;1\bar{1}} & c_{3;2\bar{3}} & c_{3;3\bar{2}} \end{array}\right|}^{+}-{\left|\begin{array}{ccc} c_{1;1\bar{2}} & c_{1;23} & c_{1;\bar{1}\;\bar{3}}\\ c_{2;1\bar{2}} & c_{2;23} & c_{2;\bar{1}\;\bar{3}}\\ c_{3;1\bar{2}} & c_{3;23} & c_{3;\bar{1}\;\bar{3}} \end{array}\right|}^{+}\\ & \;+{\left|\begin{array}{ccc} c_{1;1\bar{2}} & c_{1;2\bar{1}} & c_{1;3\bar{3}}\\ c_{2;1\bar{2}} & c_{2;2\bar{1}} & c_{2;3\bar{3}}\\ c_{3;1\bar{2}} & c_{3;2\bar{1}} & c_{3;3\bar{3}} \end{array}\right|}^{+}-{\left|\begin{array}{ccc} c_{1;1\bar{2}} & c_{1;2\bar{3}} & c_{1;3\bar{1}}\\ c_{2;1\bar{2}} & c_{2;2\bar{3}} & c_{2;3\bar{1}}\\ c_{3;1\bar{2}} & c_{3;2\bar{3}} & c_{3;3\bar{1}} \end{array}\right|}^{+}\\ & \;+{\left|\begin{array}{ccc} c_{1;1\bar{3}} & c_{1;23} & c_{1;\bar{1}\;\bar{2}}\\ c_{2;1\bar{3}} & c_{2;23} & c_{2;\bar{1}\;\bar{2}}\\ c_{3;1\bar{3}} & c_{3;23} & c_{3;\bar{1}\;\bar{2}} \end{array}\right|}^{+}-{\left|\begin{array}{ccc} c_{1;1\bar{3}} & c_{1;2\bar{1}} & c_{1;3\bar{2}}\\ c_{2;1\bar{3}} & c_{2;2\bar{1}} & c_{2;3\bar{2}}\\ c_{3;1\bar{3}} & c_{3;2\bar{1}} & c_{3;3\bar{2}} \end{array}\right|}^{+}\\ & \;+{\left|\begin{array}{ccc} c_{1;1\bar{3}} & c_{1;2\bar{2}} & c_{1;3\bar{1}}\\ c_{2;1\bar{3}} & c_{2;2\bar{2}} & c_{2;3\bar{1}}\\ c_{3;1\bar{3}} & c_{3;2\bar{2}} & c_{3;3\bar{1}} \end{array}\right|}^{+}-{\left|\begin{array}{ccc} c_{1;1\bar{3}} & c_{1;23} & c_{1;\bar{1}\;\bar{2}}\\ c_{2;1\bar{3}} & c_{2;23} & c_{2;\bar{1}\;\bar{2}}\\ c_{3;1\bar{3}} & c_{3;23} & c_{3;\bar{1}\;\bar{2}} \end{array}\right|}^{+}\\ & \;+{\left|\begin{array}{ccc} c_{1;1\bar{3}} & c_{1;2\bar{1}} & c_{1;3\bar{2}}\\ c_{2;1\bar{3}} & c_{2;2\bar{1}} & c_{2;3\bar{2}}\\ c_{3;1\bar{3}} & c_{3;2\bar{1}} & c_{3;3\bar{2}} \end{array}\right|}^{+}-{\left|\begin{array}{ccc} c_{1;1\bar{3}} & c_{1;2\bar{2}} & c_{1;3\bar{1}}\\ c_{2;1\bar{3}} & c_{2;2\bar{2}} & c_{2;3\bar{1}}\\ c_{3;1\bar{3}} & c_{3;2\bar{2}} & c_{3;3\bar{1}} \end{array}\right|}^{+} \end{array}$$

Similarly, the explicit equation for the overlap of the APsetG wavefunction with this Slater determinant is:

(A2)
$$\begin{array}{ll} & \langle 123\bar{1}\;\bar{2}\;\bar{3}|\Psi_{\text{APsetG}}\rangle =\sum_{\overset{\left\{i_{1},i_{2},i_{3}\}=\{1,2,3\right\}}{\left\{j_{1},j_{2},j_{3}\}=\{\bar{1},\bar{2},\bar{3}\right\}}}\text{sgn}\left(\sigma (i_{1},j_{1},\ldots ,i_{3},j_{3})\right)\\ & {\left|\begin{array}{ccc} c_{1;i_{1}j_{1}} & c_{1;i_{2}j_{2}} & c_{1;i_{3}j_{3}}\\ c_{2;i_{1}j_{1}} & c_{2;i_{2}j_{2}} & c_{2;i_{3}j_{3}}\\ c_{3;i_{1}j_{1}} & c_{3;i_{2}j_{2}} & c_{3;i_{3}j_{3}} \end{array}\right|}^{+}\\ & \;=-{\left|\begin{array}{ccc} c_{1;1\bar{1}} & c_{1;2\bar{2}} & c_{1;3\bar{3}}\\ c_{2;1\bar{1}} & c_{2;2\bar{2}} & c_{2;3\bar{3}}\\ c_{3;1\bar{1}} & c_{3;2\bar{2}} & c_{3;3\bar{3}} \end{array}\right|}^{+}+{\left|\begin{array}{ccc} c_{1;1\bar{1}} & c_{1;2\bar{3}} & c_{1;3\bar{2}}\\ c_{2;1\bar{1}} & c_{2;2\bar{3}} & c_{2;3\bar{2}}\\ c_{3;1\bar{1}} & c_{3;2\bar{3}} & c_{3;3\bar{2}} \end{array}\right|}^{+}\\ & \;-{\left|\begin{array}{ccc} c_{1;1\bar{2}} & c_{1;2\bar{1}} & c_{1;3\bar{3}}\\ c_{2;1\bar{2}} & c_{2;2\bar{1}} & c_{2;3\bar{3}}\\ c_{3;1\bar{2}} & c_{3;2\bar{1}} & c_{3;3\bar{3}} \end{array}\right|}^{+}+{\left|\begin{array}{ccc} c_{1;1\bar{2}} & c_{1;2\bar{3}} & c_{1;3\bar{1}}\\ c_{2;1\bar{2}} & c_{2;2\bar{3}} & c_{2;3\bar{1}}\\ c_{3;1\bar{2}} & c_{3;2\bar{3}} & c_{3;3\bar{1}} \end{array}\right|}^{+}\\ & \;-{\left|\begin{array}{ccc} c_{1;1\bar{3}} & c_{1;2\bar{1}} & c_{1;3\bar{2}}\\ c_{2;1\bar{3}} & c_{2;2\bar{1}} & c_{2;3\bar{2}}\\ c_{3;1\bar{3}} & c_{3;2\bar{1}} & c_{3;3\bar{2}} \end{array}\right|}^{+}+{\left|\begin{array}{ccc} c_{1;1\bar{3}} & c_{1;2\bar{2}} & c_{1;3\bar{1}}\\ c_{2;1\bar{3}} & c_{2;2\bar{2}} & c_{2;3\bar{1}}\\ c_{3;1\bar{3}} & c_{3;2\bar{2}} & c_{3;3\bar{1}} \end{array}\right|}^{+}\\ & \;-{\left|\begin{array}{ccc} c_{1;1\bar{3}} & c_{1;2\bar{1}} & c_{1;3\bar{2}}\\ c_{2;1\bar{3}} & c_{2;2\bar{1}} & c_{2;3\bar{2}}\\ c_{3;1\bar{3}} & c_{3;2\bar{1}} & c_{3;3\bar{2}} \end{array}\right|}^{+}+{\left|\begin{array}{ccc} c_{1;1\bar{3}} & c_{1;2\bar{2}} & c_{1;3\bar{1}}\\ c_{2;1\bar{3}} & c_{2;2\bar{2}} & c_{2;3\bar{1}}\\ c_{3;1\bar{3}} & c_{3;2\bar{2}} & c_{3;3\bar{1}} \end{array}\right|}^{+} \end{array}$$

Finally, the explicit equation for the overlap of the APIG wavefunction with this Slater determinant is:

(A3)
$$\langle 123\bar{1}\;\bar{2}\;\bar{3}|\Psi_{\text{APIG}}\rangle =-{\left|\begin{array}{lll} c_{1;1\bar{1}} & c_{1;2\bar{2}} & c_{1;3\bar{3}}\\ c_{2;1\bar{1}} & c_{2;2\bar{2}} & c_{2;3\bar{3}}\\ c_{3;1\bar{1}} & c_{3;2\bar{2}} & c_{3;3\bar{3}} \end{array}\right|}^{+}$$

## Appendix B Proof for Equation (12)

The proof is by induction. Starting with the 2×2 permanent:

(B1)
$$\begin{array}{ll} \text{abs}({\left|\begin{array}{cc} c_{11} & c_{12}\\ c_{21} & c_{22} \end{array}\right|}^{+}) & =|c_{11}c_{22}+c_{12}c_{21}|\\ & \leq |c_{11}c_{22}|+|c_{12}c_{21}|+|c_{11}c_{12}|+|c_{21}c_{22}|\\ & =(|c_{11}|+|c_{21}|)(|c_{12}|+|c_{22}|) \end{array}$$

The inequality is also true for the 3×3 permanent:

(B2)
$$\begin{array}{ll} & \text{abs}\left({\left|\begin{array}{ccc} c_{11} & c_{12} & c_{13}\\ c_{21} & c_{22} & c_{23}\\ c_{31} & c_{32} & c_{33} \end{array}\right|}^{+}\right)=|c_{11}|\cdot \text{abs}\left({\left|\begin{array}{cc} c_{22} & c_{23}\\ c_{32} & c_{33} \end{array}\right|}^{+}\right)\\ & +|c_{21}|\cdot \text{abs}\left({\left|\begin{array}{cc} c_{12} & c_{13}\\ c_{32} & c_{33} \end{array}\right|}^{+}\right)+|c_{31}|\cdot \text{abs}\left({\left|\begin{array}{cc} c_{12} & c_{13}\\ c_{22} & c_{23} \end{array}\right|}^{+}\right)\\ & \leq (|c_{11}|+|c_{21}|+|c_{31}|)\;\max(\text{abs}({\left|\begin{array}{cc} c_{22} & c_{23}\\ c_{32} & c_{33} \end{array}\right|}^{+})\\ & \text{abs}({\left|\begin{array}{cc} c_{12} & c_{13}\\ c_{32} & c_{33} \end{array}\right|}^{+}),\text{abs}({\left|\begin{array}{cc} c_{12} & c_{13}\\ c_{22} & c_{23} \end{array}\right|}^{+}))\\ & \leq (|c_{11}|+|c_{21}|+|c_{31}|)\\ & \;\cdot \max((|c_{22}|+|c_{32}|)(|c_{23}|+|c_{33}|),(|c_{12}|+|c_{32}|)(|c_{13}|+|c_{33}|)\\ & \left(|c_{12}|+|c_{22}|)(|c_{13}|+|c_{23}|)\right)\\ & \leq (|c_{11}|+|c_{21}|+|c_{31}|)(|c_{12}|+|c_{22}|+|c_{32}|)(|c_{13}|+|c_{23}|+|c_{33}|) \end{array}$$

Using the same argument, we can prove the theorem by induction. We use the Laplace expansion of the permanent,

(B3)
$$|C{|}^{+}=\overset{P}{\underset{i=1}{\sum }}\;c_{i1}|M_{i1}{|}^{+}$$

where $M_{ij}$ is the minor of A where the row i and column 1 were removed. Then,

(B4)
$$\begin{array}{ll} \text{abs}(|C{|}^{+}) & =\overset{P}{\underset{i=1}{\sum }}|c_{i1}|\cdot \text{abs}(|M_{i1}{|}^{+})\\ & \leq \left(\overset{P}{\underset{i=1}{\sum }}|c_{i1}|\right)\max_{i}\left(\text{abs}(M_{i1})\right)\\ & \leq \left(\overset{P}{\underset{i=1}{\sum }}|c_{i1}|\right)\max_{i}(\overset{P}{\underset{j=2}{\prod }}\;\overset{P}{\underset{k\neq i}{\sum }}|c_{kj}|)\\ & \leq \left(\overset{P}{\underset{i=1}{\sum }}|c_{i1}|\right)\left(\overset{P}{\underset{j=2}{\prod }}\;\overset{P}{\underset{i=1}{\sum }}|c_{ij}|\right)\\ & \leq \overset{P}{\underset{j=1}{\prod }}\;\overset{P}{\underset{i=1}{\sum }}|c_{ij}| \end{array}$$

In the third line of the derivation we used the (previously derived) inequality for the (P−1)×(P−1) permanent. Inspired by the derivation for the 3×3 case, in the fourth line, we (a) added an extra positive term, $\left|c_{ki}\right|$, to each term in the innermost sum and then (b) noticed that all the terms that were being maximized over were identical, so that the maximization was unnecessary.
